# Myelin modulates the process of isoflurane anesthesia through the regulation of neural activity

**DOI:** 10.1111/cns.14922

**Published:** 2024-08-13

**Authors:** Xu Wang, Rulan Yi, Xiaoling Liang, Ning Zhang, Fuwang Zhong, Yali Lu, Wenjia Chen, Tian Yu, Linyong Zhang, Haiying Wang, Liang Zhou

**Affiliations:** ^1^ Key Laboratory of Anesthesia and Organ Protection (Zunyi Medical University), Ministry of Education Zunyi Medical University Zunyi China; ^2^ Key Laboratory of Brain Science Zunyi Medical University Zunyi China; ^3^ Guizhou Key Laboratory of Anesthesia and Organ Protection Zunyi Medical University Zunyi China; ^4^ Department of Anesthesiology Affiliated Hospital of Zunyi Medical University Zunyi China

**Keywords:** consciousness, general anesthesia, isoflurane, myelin, neural activity

## Abstract

**Aims:**

The mechanism underlying the reversible unconsciousness induced by general anesthetics (GA) remains unclear. Recent studies revealed the critical roles of myelin and oligodendrocytes (OLs) in higher functions of the brain. However, it is unknown whether myelin actively participates in the regulation of GA. The aim of this study is to investigate the roles and possible mechanisms of myelin in the regulation of consciousness alterations induced by isoflurane anesthesia.

**Methods:**

First, demyelination models for the entire brain and specific neural nuclei were established to investigate the potential role of myelination in the regulation of GA, as well as its possible regional specificity. c‐Fos staining was then performed on the demyelinated nuclei to verify the impact of myelin loss on neuronal activity. Finally, the activity of neurons during isoflurane anesthesia in demyelinated mice was recorded by optical fiber photometric calcium signal. The related behavioral indicators and EEG were recorded and analyzed.

**Results:**

A prolonged emergence time was observed from isoflurane anesthesia in demyelinated mice, which suggested the involvement of myelin in regulating GA. The demyelination in distinct nuclei by LPC further clarified the region‐specific roles of isoflurane anesthesia regulation by myelin. The effect of demyelination on isoflurane anesthesia in the certain nucleus was consistent with that in neurons towards isoflurane anesthesia. Finally, we found that the mechanism of myelin in the modulation of isoflurane anesthesia is possibly through the regulation of neuronal activity.

**Conclusions:**

In brief, myelin in the distinct neural nucleus plays an essential role in regulating the process of isoflurane anesthesia. The possible mechanism of myelin in the regulation of isoflurane anesthesia is neuronal activity modification by myelin integrity during GA. Our findings enhanced the comprehension of myelin function, and offered a fresh perspective for investigating the neural mechanisms of GA.

## Graphical abstract

Myelin in the distinct neural nucleus plays an essential role in regulating the progress of isoflurane anesthesia. The possible mechanism of myelin in the regulation of isoflurane anesthesia is the modification of neuronal activity by myelin integrity during GA.

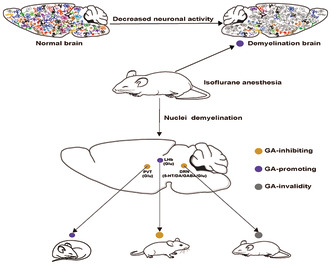

## 1 INTRODUCTION

General anesthesia (GA) is a state of reversible consciousness suppression, amnesia, analgesia, sedation and immobility induced by anesthetics.^1^, ^2^, ^3^ The application of GA has significantly advanced the development and progression of human surgical medicine; however, the mechanism underlying reversible unconsciousness induced by GA remains unclear. Former studies were focused on the roles of neural circuits in GA. Several neural nuclei have been identified to involve in regulating GA mediated unconsciousness, including the thalamic reticular nucleus (TRN) and the lateral habenula (LHb), which contribute to anesthesia induction, as well as the paraventricular nucleus (PVT), the dorsal raphe nucleus (DRN) and the ventral tegmental area (VTA), which facilitate wakefulness from anesthesia.^4^, ^5^, ^6^, ^7^, ^8^, ^9^, ^10^ Furthermore, the functions and mechanisms of the certain type of neurons and the related neural circuits of these distinct nuclei in GA modulation have been clarified.^11^, ^12^, ^13^, ^14^ Recently, the participation of microglia and astrocyte in the regulation of GA has been reported.^3^, ^15^, ^16^, ^17^, ^18^ These studies expanded and deepened the recognition of the neural mechanisms of GA. However, the involvement of other types of neural cells and structures, such as oligodendrocytes (OLs) formed myelin, has been poorly investigated.

The myelin, a lipid membrane structure formed by oligodendrocytes (OLs) wrapping the axons of neurons in the central nervous system (CNS), plays crucial roles in insulating, protecting, providing nutritional support for axons, and ensuring rapid and precise transmission of nerve electrical signals.^19^, ^20^, ^21^, ^22^ The structural damages or developmental defects of myelin disrupt the transmission of electrical signals, leading to fundamental functional impairments in neurons. As a result, myelin disruption affects neural circuits involved in higher‐order brain functions such as emotion, cognition, learning and memory, as well as motor behavior.^23^, ^24^, ^25^ These studies suggest that the myelin integrity may be crucial for higher‐order neural functions, such as consciousness and cognition.

An increasing number of neural circuits in the regulation of consciousness changes induced by GA has been identified, whereas the myelin integrity is crucial for normal function of neural circuits. Therefore, we speculate the possible involvement of myelin in GA modulation. On one hand, numerous studies have demonstrated that GA can induce abnormal myelin development in mammals and rodents, resulting in cognitive impairments, learning and memory deficits, fine motor dysfunction, and other related disorders.^26^, ^27^, ^28^, ^29^, ^30^ On the other hand, myelin has been proved to actively involve in multiple higher‐order brain function modification.^31^, ^32^ Notably, myelin repair has been developed as an important topic for many brain diseases.^33^, ^34^ Therefore, investigating the role of myelin in regulation of GA offers a fresh perspective and a potential target for the neural mechanism of GA.

To investigate this hypothesis, we initially anesthetized cuprizone‐induced demyelination mice with isoflurane and observed a significant delay in their emergence, thus providing evidence for the involvement of myelin in the process of GA. Recent studies have shown that an aberrant loss of myelin in the PVT results in anxiety‐like emotions, which can be alleviated through myelin restoration, indicating the regulatory influence of the myelin in specific nucleus on higher brain functions.^35^ Given that the PVT was identified as one of the most important emergence‐promoting brain regions during GA,^6^, ^9^ we suggested that myelin in the PVT may also affected the anesthesia progression. Different nuclei exert distinct effects on the induction and emergence of GA.^4^ We induced demyelination in the distinct neural nuclei that have been proven to be involved in the regulation of GA to test whether the myelin in the specific brain regions can modulate GA process. Indeed, we observed that myelin modulates GA in a region‐specific manner. Then we conducted c‐Fos staining and calcium fiber photometry recordings to observe the impact of myelin defect on neuronal activity in these nuclei, which revealed a significant reduction in neuronal activity following demyelination and lack of response to the stimulatory effects of isoflurane. It is suggested that myelin may be involved in the regulation of consciousness change induced by GA via affecting the activity of neurons. Taken together, our study demonstrates the regulatory role of myelin in GA and identifies a possible underlying mechanism. It provides a new perspective to elucidate the mechanism of GA and extends the roles of myelin in the higher‐order functions of brain.

## 2 MATERIALS AND METHODS

### 2.1 Animals

This study was performed in accordance with the Guide for the Care and Use of Laboratory Animals of China (no. 14924, 2001). All animal procedures were designed and conducted according to the guidelines set forth by the Zunyi Medical University. Male C57BL/6J mice (7–15 weeks old, weighing 20–30 g) were used for all experiments and purchased from Jiangsu Huachuang Cigna Medical Technology Co., Ltd. (Taizhou, Jiangsu, China). All animals were housed in the Laboratory Animal Center of Zunyi Medical University, with a constant temperature of 24°C (±1°C) and a relative humidity of 55% (±2%) on a 12:12 h light/dark cycle (lights on at 6:00 am), while having free access to food and water. To minimize the impact of room temperature on experimental results, all behavioral and electroencephalogram (EEG) tests were conducted at a controlled temperature of 24°C (±2°C).

### 2.2 Antibodies and reagents

The primary antibodies against MBP (MAB386, RRID: AB_94975), and NeuN (MAB377, RRID: AB_2298772) were obtained from Millipore (Billerica, MA, USA). c‐Fos antibody (226008, RRID: AB_2891278) was from Synaptic Systems (Goettingen, Germany). 5‐HT antibody (ab211528, RRID: AB_3344910) was from Abcam (Cambridge, UK). Alexa Fluor‐conjugated secondary antibodies (A‐11039, RRID: AB_142924; A‐11008, RRID: AB_143165; and A‐11005, RRID: AB_141372) were purchased from Invitrogen (Carlsbad, CA, USA). Lysolecithin (LPC, L4129) was from Sigma (TUCSON, AZ, USA). Ibotenic acid (IBO, HY‐N2311) was purchased from MedChemExpress LLC (USA).

### 2.3 Surgery

The mice were anesthetized with isoflurane throughout, secured in a stereotaxic apparatus (RWD Life Science Co. Ltd., Shenzhen China), and their eyes were covered with erythromycin eye ointment. The scalp was cut to expose the skull, and the level of the skull was adjusted until leveled. According to the mouse brain stereotaxic atlas (Watson and Paxinos), the Bregma serves as the origin with mm as the unit, and specific brain regions are located using the following stereotaxic coordinates [anterior–posterior (AP), medial‐lateral (ML), dorsal‐ventral (DV)]: the lateral habenula (LHb; AP—1.7, ML ± 0.45, DV—2.85), the paraventricular nucleus (PVT; AP—1.3, ML ± 0.0, DV—3.0), the dorsal raphe nucleus (DRN; AP—4.6, ML ± 0.0, DV—3.05). Use a microsyringe pump (Oran Science and Technology Co., Ltd., USA) to inject drugs or viruses through a glass microelectrode (Taimeng Software Co., Ltd., Chengdu, Sichuan, China). After injection completed, retain the microelectrode at the injection site for 10 min to ensure full diffusion of the drugs or the viruses before gradually withdrawn.

For LPC induced demyelination experiments, LPC (10 mg/mL) was injected into LHb (0.5 μL/side), PVT (1 μL) and DRN (1 μL) at a rate of 100 nL / min to generate the LPC induced demyelination model (LPC group), and the same dose of normal saline (NS) was injected into control group (control group). After drug injection, four screws were inserted into the skull (AP: + 1.0 mm, ML: ± 1.5 mm; AP: − 3.5 mm, ML: ± 1 mm). The EEG electrodes were placed and secured with dental cement and glue. Behavioral experiments and EEG recording were conducted after 7 days.

For neuronal lesion experiments, IBO was injected into LHb (100 nL/side), PVT (200 nL) and DRN (200 nL) at a rate of 20 nL/min to establish the IBO group, and the same dose of NS was injected into the control group. Behavioral experiments were conducted after 7 days.

For in vivo fiber calcium imaging experiment in the demyelinated mice, LPC (10 mg/mL) was injected into LHb (0.5 μL/side), PVT (1 μL) and DRN (1 μL) at a rate of 100 nL/min, and the same dose of NS was injected into the control group. Then the GCaM6s viruses were injected into LHb (50 nL), PVT (80 nL), and DRN (80 nL), respectively (LHb and PVT: rAAV‐CaMKIIα‐GCaM6s; DRN: rAAV‐hsyn‐SV40‐NLS‐Cre and rAAV‐EF1α‐DIO‐GCaMP6s) (BrainVTA Co., Ltd., Wuhan, Hubei, China). Finally, the optical fiber (Inper Ltd, Hangzhou, Zhejiang, China) was inserted 0.1 mm shallower than the injection sites. Calcium recordings were carried out after 3 weeks.

For chemogenetic activation experiment in the demyelinated mice, LPC (10 mg/mL) was injected into LHb (0.5 μL/side), PVT (1 μL) and DRN (1 μL) at a rate of 100 nL/min to generate the demyelination model, and the same dose of NS was injected into the control group. Then the hM3D(Gq) viruses were injected into LHb (50 nL), PVT (80 nL), and DRN (80 nL), respectively (LHb and PVT: rAAV‐CaMKIIα‐hM3D(Gq); DRN: rAAV‐hsyn‐SV40‐NLS‐Cre and rAAV‐EF1α‐DIO‐ hM3D(Gq)) (BrainVTA Co., Ltd., Wuhan, Hubei China). Behavioral experiments were conducted after 3 weeks.

### 2.4 Cuprizone (CPZ) induced demyelination

For effective demyelination, 7‐week‐old C57BL/6J male mice were fed with 0.3% CPZ (C9012, Sigma, TUCSON, AZ, USA) diet for 4 consecutive weeks to establish a demyelination model. The control groups were fed with routine diet.

### 2.5 Behavioral tests of isoflurane anesthesia

The time taken from the initiation of anesthesia to loss of righting reflex (LORR) and from the termination of anesthesia to recovery of righting reflex (RORR) were utilized for quantifying the induction and the emergence time of anesthesia, respectively. The mice were gently placed in an induction box (RWD Life Science Co. Ltd., Shenzhen China), acclimated for 5 min, and then the inhalation of 1 L/min of 1% or 1.4% isoflurane with 100% O_2_ was initiated to measure the LORR time. After a 20‐min maintenance of isoflurane inhalation, the isoflurane administration was discontinued and the RORR time was recorded. An anesthesia monitor (Vamos; Drager Company, Germany) was connected to detect the concentration of isoflurane in the anesthesia chamber and an electric blanket with a rectal temperature probe was used to the bottom of the anesthesia chamber and was controlled at 37.5°C in the whole experiment. The mice were removed after recording, then the induction box was cleaned with 75% alcohol to remove odors. All mice were placed in the behavioral room at least 1 h in advance.

### 2.6 EEG recording

Electroencephalogram signals were collected using an Apollo II multi‐channel neural signal data acquisition system (Bio‐Signal Technologies, Nanjing, Jiangsu, China). EEG signals were recorded for 5 min before induction and were continuously recorded until recovery from isoflurane anesthesia. Then, the Spike2 software (Cambridge Electronic Design Limited Technical Centre, Cambridgeshire, England, UK) was used to analyze the percentage of power in different frequency bands of EEG during different periods. The frequency bands can be divided as follows: delta (𝛿): 1 ~ 4 Hz, theta (𝜃): 4 ~ 8 Hz, alpha (α): 8 ~ 12 Hz, beta (𝛽): 12 ~ 25 Hz, and gamma (𝛾): 25 ~ 60 Hz. Finally, the NeuroExplorer software (Nex Technology, Littleton, USA) was utilized to generate EEG power spectrogram.

### 2.7 Calcium fiber photometry

Fluorescence signal of GCaMP6s was acquired using a dual‐color fiber recording device (Inper Ltd, Hangzhou, Zhejiang, China) with a sampling frequency of 100 Hz, an exposure time of 10 ms, and a gain setting of 0. The 410 nm channel was used as the reference channel, while the 470 nm channel was utilized for recording calcium signals. The fiber output power of both channels was regulated within the range of 10–20 μW and 20–40 μW, respectively. The mice were placed in the dark experimental room for a one‐h acclimation period prior to the experiment. Following 5 min of baseline recording after adaption in the induction box, isoflurane was continuously delivered for 10 min. The recording was continued for another 5 min after turning off the isoflurane. The data were analyzed by InperDataProcessV0.7.2 software (Inper Ltd., Hangzhou, Zhejiang, China). We first preprocessed the original data, including baseline correction and marker increase and decrease before further analyzing the data to obtain the heat map and linear map. Furthermore, ∆*F*/*F* was analyzed [(*F* − *F*0)/*F* = ∆*F*/*F*, was the change of original fluorescence signal (*F*) relative to *F*0; *F*, original fluorescence signal; *F*0, the mean value of original fluorescence signal]. The Δ*F*/*F* were then compared between the LPC and Control groups during each period.

### 2.8 Immunohistochemistry

After all tests completed, mice were anesthetized, sacrificed and fixed. The heart was exposed, and the blood was washed out by rapid perfusion through the heart with phosphate‐buffered saline (PBS). When the liver turned white, 4% Paraformaldehyde (PFA) was continuously injected for fixation. After perfusion, the whole brain was dissected out and soaked in 4% PFA for 24 h. Then the brain was dehydrated by 30% sucrose. Brain tissues were frozen in OCT and sectioned with a thickness of 30 μm using a cryostat microtome (Leica CM1900, Germany). The expression of MBP, NeuN, 5‐HT, and calcium signal virus in brain slices were observed using immunofluorescence staining. The main procedures were as follows: brain slices were blocked with blocking buffer (10% goat serum, 1% BSA, and 0.3% Triton in PBS) for 2 h at room temperature. The primary antibodies were incubated overnight at 4°C. Then the secondary antibodies were then incubated for 2 h at room temperature in the dark. The dilution ratios of primary antibodies were 1:500 for MBP; 1:200 for NeuN and 5‐HT; and 1:1000 for c‐Fos and the secondary antibodies were diluted at 1:500. Finally, the nuclei were stained with DAPI Fluoromount‐G Reagent (SBA‐0100‐20, Southern Biotech, Birmingham, AL, USA). Immunofluorescence images were captured using a fluorescence microscope (Olympus, Tokyo, Japan) and subsequently analyzed with ImageJ 1.42q (National Institutes of Health, Bethesda, MD, USA).

### 2.9 Statistical analysis

Statistical analysis was performed using SPSS 29.0 (IBM Corp., Armonk, NY, USA, Version 29.0), and the data are presented as mean ± SD. The two groups were compared using an unpaired Student's *t*‐test. The effect of LPC‐induced demyelination on neuron activation by chemogenetics during isoflurane anesthesia was analyzed by two‐way ANOVA. GraphPad Prism 8.0 software (GraphPad Software Inc., San Diego, CA, USA) was used for statistical chart production after data analysis. *p* < 0.05 was considered statistically significant, and “n” represents the number of animals used in the corresponding experiment.

## 3 RESULTS

### 3.1 Cuprizone‐induced demyelination delays the emergence from isoflurane anesthesia

**FIGURE 1:**
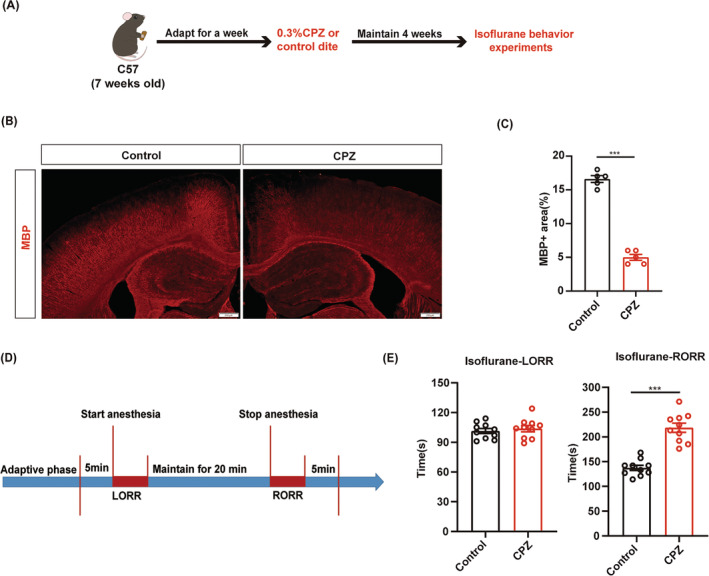
Cuprizone‐induced myelination delays the emergency from isoflurane anesthesia. (A) The experimental scheme for establishing a CPZ‐induced demyelination model and conducting behavioral experiments. (B) Representative images of MBP expression (red) in CPZ and control groups. Scale bar; 100 μm. (C) Quantification of MBP+ area in the control and CPZ groups. (D) Flow chart for behavioral experiments during 1.4% isoflurane anesthesia. (E) Bar graphs of LORR and RORR time measurement under 1.4% isoflurane anesthesia. in the CPZ and control groups (*n* = 10/group). ***p < 0.001, unpaired Student's *t*‐test. CPZ, cuprizone; MBP, myelin basic protein; LORR, loss of righting reflex; RORR, recovery of righting reflex.

To investigate the role of myelin in isoflurane anesthesia, we first induced demyelination in the CNS of C57BL/6J male mice through continuous feeding of a diet containing cuprizone (CPZ) (Figure 1A). After 4 weeks, MBP expression was significantly reduced in the CPZ group compared to the control group, which indicates successful establishment of the demyelination model (Figure 1B,C). Behavioral tests were conducted under 1.4% isoflurane anesthesia (Figure 1D). The results showed a prolonged RORR time in the CPZ group compared to the control group, while the LORR time was comparable between the CPZ and control groups (Figure 1E). These data indicated that myelin destruction in the CNS may affect the process of isoflurane anesthesia.

### 3.2 LHb demyelination promotes the emergence from isoflurane anesthesia

**FIGURE 2:**
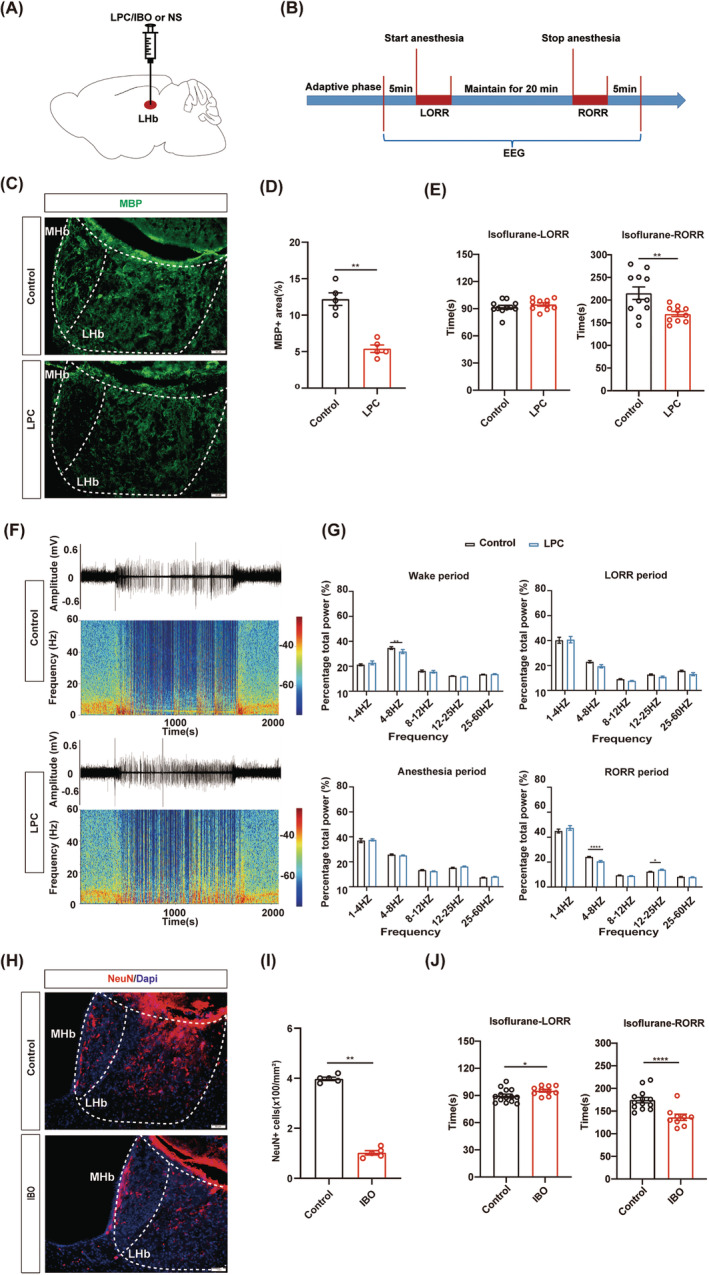
LHb demyelination promotes emergence from isoflurane anesthesia. (A) Schematic of drug injection. (B) Flow chart for behavioral and EEG recording under isoflurane anesthesia. (C) Representative images of MBP staining (green) in the LHb of the LPC and control groups. Scale bar; 50 μm. (D) Quantification of MBP+ area in the LHb of control and LPC groups. (E) Bar graphs of LORR and RORR time in LPC group and control group during 1.4% isoflurane anesthesia (*n* = 10 in the LPC group, *n* = 11 in the control group). (F) Representative raw EEG traces and corresponding power spectrograms of the control group (top) versus the LPC group (bottom) during 1.4% isoflurane anesthesia. (G) The percentage of EEG power in different frequency bands during various periods of isoflurane anesthesia was compared between the LPC group and control groups (*n* = 10 in the LPC group, *n* = 11 in the control group). (H) Representative images of NeuN staining (red) in IBO and control groups. Scale bar; 50 μm. (I) Quantification of NeuN+ number (mm^2^) in the LHb of control and IBO groups. (J) Bar graphs of LORR and RORR time in IBO group and control groups during 1.4% isoflurane anesthesia (*n* = 10 in the IBO group, *n* = 14 in the control group). *p < 0.05, **p < 0.01, ****p < 0.0001, unpaired Student's *t*‐test. LPC, lysolecithin; IBO, Ibotenic acid; LHb, the lateral habenula; NeuN, neuronal nuclei.

Recent research has revealed that myelin in specific nuclei regulate higher‐order brain functions.^35^ However, whether myelin in the distinct nucleus is involved in the regulation of isoflurane anesthesia remains unknown. LHb was reported to promote the induction of general anesthesia.^36^ So, we wonder that myelin in the LHb can participate in the progression of isoflurane anesthesia. We bilaterally injected LPC (the LPC group) or saline (the control group) into the LHb to generate demyelination in the LHb (Figure 2A). After 1 week, dramatically decreased MBP expression in the LHb of the LPC group indicated the myelin destruction in the LHb (Figure 2C,D). In the behavior test of 1.4% isoflurane anesthesia, we found that the RORR time in the LPC group was shorter compared to the control group, while no significant difference was observed in the LORR time (Figure 2B,E). These findings suggest that demyelination in the LHb promotes the emergence from isoflurane anesthesia. The EEG results were consistent with the behavioral results. Compared to the control group, the power in the theta band was decreased and the power in the beta band was increased in the LPC group during RORR (Figure 2F,G). Notably, the power of theta band in the LPC group was lower than that of the control group during the WAKE phase prior to anesthesia (Figure 2G). Similarly, analysis of both raw EEG traces and EEG power spectrograms indicated higher levels of activity and wakefulness in the LPC group compared to the control group (Figure 2F). Additionally, we performed the behavior test under 1% isoflurane anesthesia and found that demyelination in the LHb also promoted the emergence from 1% isoflurane anesthesia (Figure S1A,B,E).

Next, we compared the effects of myelin damage and neuron lesion in the LHb on isoflurane anesthesia. IBO (the IBO group) or saline (the control group) was bilaterally injected into the LHb region to kill the LHb neurons (Figure 2H,I). During 1.4% isoflurane anesthesia, the LORR time was prolonged and the RORR time was shortened in the IBO group (Figure 2J). This suggests that depletion of LHb neurons inhibits the anesthetic effect of isoflurane. Taken together, we conclude that both demyelination and neuronal depletion in the LHb contribute to emergence from anesthesia.

### 3.3 PVT demyelination facilitates the induction of isoflurane anesthesia

**FIGURE 3:**
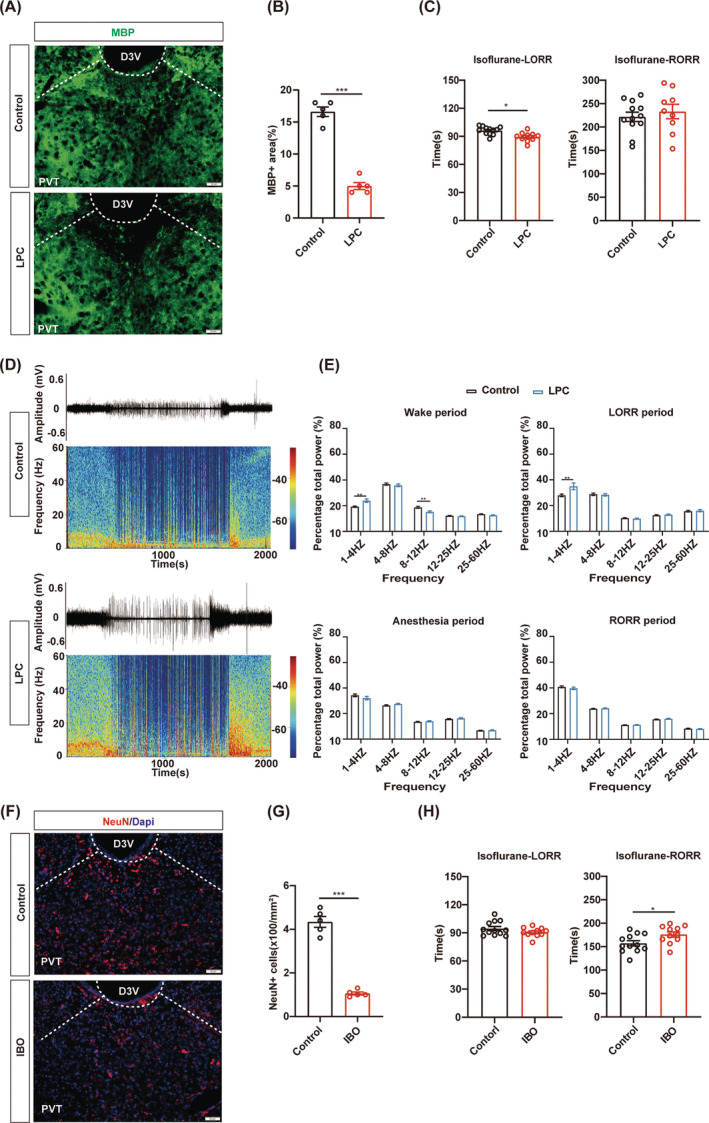
Paraventricular nucleus demyelination facilitates the induction of isoflurane anesthesia. (A) Representative image of MBP staining (green) in the PVT of the LPC and control groups. Scale bar; 50 μm. (B) Quantification of MBP+ area in the PVT of control and LPC groups. (C) Bar graphs of LORR and RORR time in LPC group and control group during 1.4% isoflurane anesthesia (*n* = 10 in the LPC group, *n* = 12 in the control group). (D) Representative raw EEG traces and corresponding power spectrograms of the control group (top) versus the LPC group (bottom) during 1.4% isoflurane anesthesia. (E) The percentage of EEG power in different frequency bands during various periods of isoflurane anesthesia was compared between the LPC group and control group. (F) Representative images of NeuN staining (red) after IBO‐induced depletion of PVT neurons. Scale bar; 50 μm. (G) Quantification of NeuN+ number (mm^2^) in the PVT of control and IBO groups. (H) Bar graphs of LORR and RORR time in IBO and control groups during 1.4% isoflurane anesthesia (*n* = 11 in the IBO group, *n* = 12 in the Control group). *p < 0.05, **p < 0.01, ***p < 0.001, unpaired Student's *t* test. PVT, the paraventricular nucleus.

Next, we selected the PVT, which has been demonstrated to plays a crucial role in facilitating recovery from anesthesia,^9^ to study whether myelin in the PVT is involved in the regulation of isoflurane anesthesia. LPC was injected into the PVT to induce the demyelination. MBP expression in the PVT was reduced in the LPC group compared to the control group (Figure 3A,B). The behavioral tests of 1.4% isoflurane anesthesia showed the shortened LORR time in the LPC group and comparable RORR time between the LPC group and the control group (Figure 3C). The EEG recording revealed that the LPC group exhibited a higher power of the delta band compared to the control group during LORR, suggesting an enhanced effect of isoflurane anesthesia following PVT demyelination (Figure 3E). Compared with the control group, the power of the delta band was increased and the power of the alpha band was decreased in the LPC group during the WAKE period, suggesting that PVT demyelination reduced awaken activity of EEG in mice (Figure 3E). From the raw EEG traces and EEG power spectrograms, it can also be observed that mice in the LPC group transitioned more easily from an awake state to an anesthesia state. Additionally, the overall excitability of cortical brain electrical activity was markedly lower in the LPC group compared to the control group, indicating that demyelination of the PVT puts a sedation‐like state in mice (Figure 3D). Furthermore, the LPC group showed shortened LORR time and prolonged RORR time during 1% isoflurane anesthesia (Figure S1A,C,F).

IBO‐induced neuronal depletion in the PVT was also performed to compare the effects of myelin and neurons in the PVT on isoflurane anesthesia (Figure 3F,G). Normal LORR time and prolonged RORR time under 1.4% isoflurane anesthesia were observed in the IBO group, indicating that the arousal‐promotion effect of the PVT was suppressed by neuron depletion (Figure 3H). It is suggested that both demyelination and neuronal depletion in the PVT contribute to the action of isoflurane, whereas the process may differ. These findings suggest that the myelin in specific nuclei may play an essential role in regulating consciousness change during isoflurane anesthesia. Furthermore, our data also indicated that the mechanisms underlying general anesthesia may differ between processes involving the myelin and neurons, even in the same nucleus.

### 3.4 The effect of isoflurane anesthesia is not affected by DRN demyelination

**FIGURE 4:**
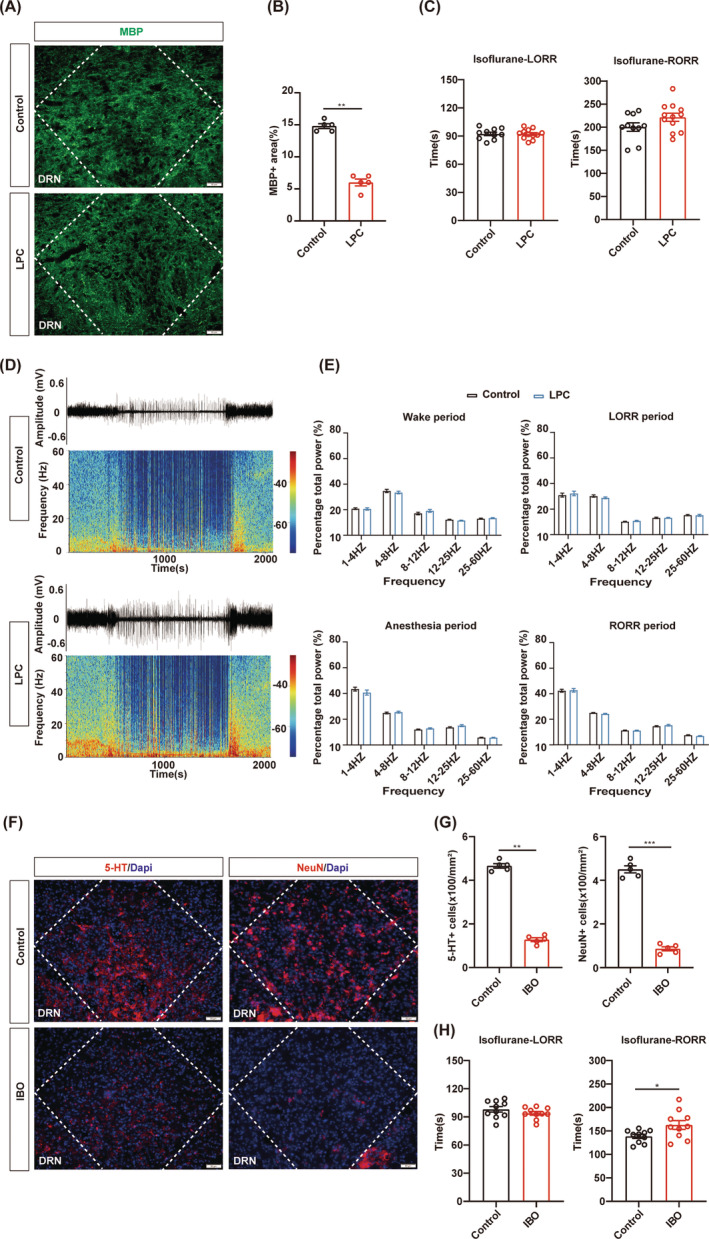
The effect of DRN demyelination on isoflurane anesthesia. (A) Representative image of MBP staining (green) in the DRN of the LPC and control groups. Scale bar; 50 μm. (B) Quantification of MBP+ area in the DRN of control and LPC groups. (C) Bar graphs of LORR and RORR time in LPC group and control group during 1.4% isoflurane anesthesia (*n* = 12 in the LPC group, *n* = 10 in the control group). (D) Representative raw EEG traces and corresponding power spectrograms of the control group (top) versus the LPC group (bottom) during 1.4% isoflurane anesthesia. (E) The percentage of EEG power in different frequency bands during various periods of isoflurane anesthesia was compared between the LPC group and control group. (F) Representative images (red) of 5‐HT (top) and NeuN (bottom) after IBO‐induced depletion of DRN neurons. Scale bar; 50 μm. (G) Quantification of 5‐HT+ and NeuN+ number (mm^2^) in the DRN of control and IBO groups. (H) Bar graphs of LORR and RORR time in IBO group and control group during 1.4% isoflurane anesthesia (*n* = 10 in the IBO group, *n* = 10 in the control group). *p < 0.05, **p < 0.01, ***p < 0.001, unpaired Student's *t*‐test. DRN, the dorsal raphe nucleus; 5‐HT, 5‐Hydroxytryptamine.

Interestingly, both LHb and PVT are mainly composed of a single type of neurons. The potential reason behind our findings may be attributed to the neuronal homogeneity within the nucleus, as their specific influence on GA is clearly defined. Given the distinct roles of different types of neurons in the nucleus with a diversity of neuron types, we were intrigued by the impact of demyelination in heterogeneous nuclei on the modulation of general anesthesia. The dorsal raphe nucleus (DRN), which is composed of diverse subtypes of neurons, plays multiple roles in general anesthesia, making it the target of our study.^37^, ^38^, ^39^, ^40^ LPC injection was performed to induce demyelination in the DRN and the decrease MBP expression confirmed the efficiency of myelin defect in the DRN (Figure 4A,B). The LPC group and the control group showed the comparable LORR and RORR time during 1.4% isoflurane anesthesia, indicating that DRN demyelination did not affect the progress of isoflurane anesthesia (Figure 4C). There was no significant difference in the percentage of EEG power in different frequency bands between the two groups, which is consistent with the behavioral results (Figure 4D,E). Correspondingly, demyelination in the DRN exhibited no significant effect on LORR or RORR time during 1% isoflurane anesthesia (Figure S1A,D,G).

Furthermore, IBO‐induced neuron deletion in the DRN resulted in a significant reduction in both 5‐HT and NeuN neuron populations (Figure 4F,G). The RORR time was prolonged in the IBO group under 1.4% isoflurane anesthesia, but there was no difference in LORR time between the two groups (Figure 4H). These findings are consistent with the former study that reported the arousal effect of DRN in GA.^41^, ^42^ Together, demyelination in the DRN did not affect the anesthetic effect, while neuron depletion in the DRN enhanced the anesthetic effect.

### 3.5 Demyelination causes decreased neuronal activity in the awake mice

**FIGURE 5:**
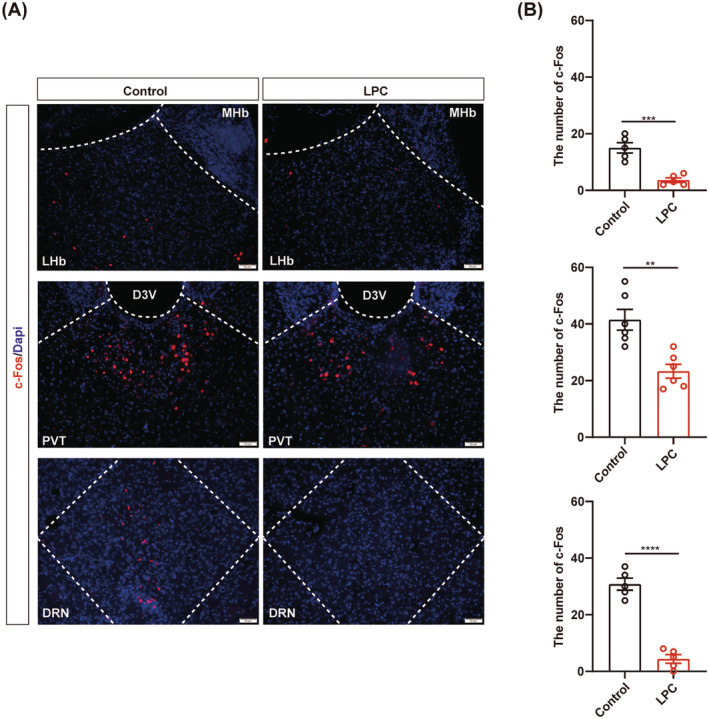
Decreased expression of c‐Fos in the LHb, PVT and DRN after demyelination of the awaking mice, respectively. (A) Representative image of c‐Fos expression (red) in the LHb (top), PVT (middle) and DRN (bottom) demyelination groups and control groups during the awaking stage, respectively. Scale bar; 50 μm. (B) Bar graphs of c‐Fos number in the LHb (top), PVT (middle), and DRN (bottom) during the awaking stage in the LPC and control groups (*n* = 5/group). **p < 0.01, ***p < 0.001, ****p < 0.0001, unpaired Student's *t*‐test. LHb, the lateral habenula; PVT, the paraventricular nucleus; DRN, the dorsal raphe nucleus.

Overall, our findings demonstrate that myelin in the certain nuclei participate in the regulation of isoflurane anesthesia, although its' specific effect may differ from the roles of neurons in the corresponding nucleus. The different roles of myelin and neurons in GA modulation may due to the effect of myelin destruction on neuronal activity. To determine the relationship between demyelination and neuronal activity in the corresponding nucleus, we set up c‐Fos straining to monitor the neural activity after demyelination in the LHb, PVT and DRN, respectively. We found decreased c‐Fos in the awake mice after demyelination (Figure 5A,B), demonstrating that demyelination reduces neuronal activity, which suggested that myelin may be involved in the regulation of GA, potentially by influencing neuronal activity.

### 3.6 Myelin loss causes failure response of the neurons to GA

**FIGURE 6:**
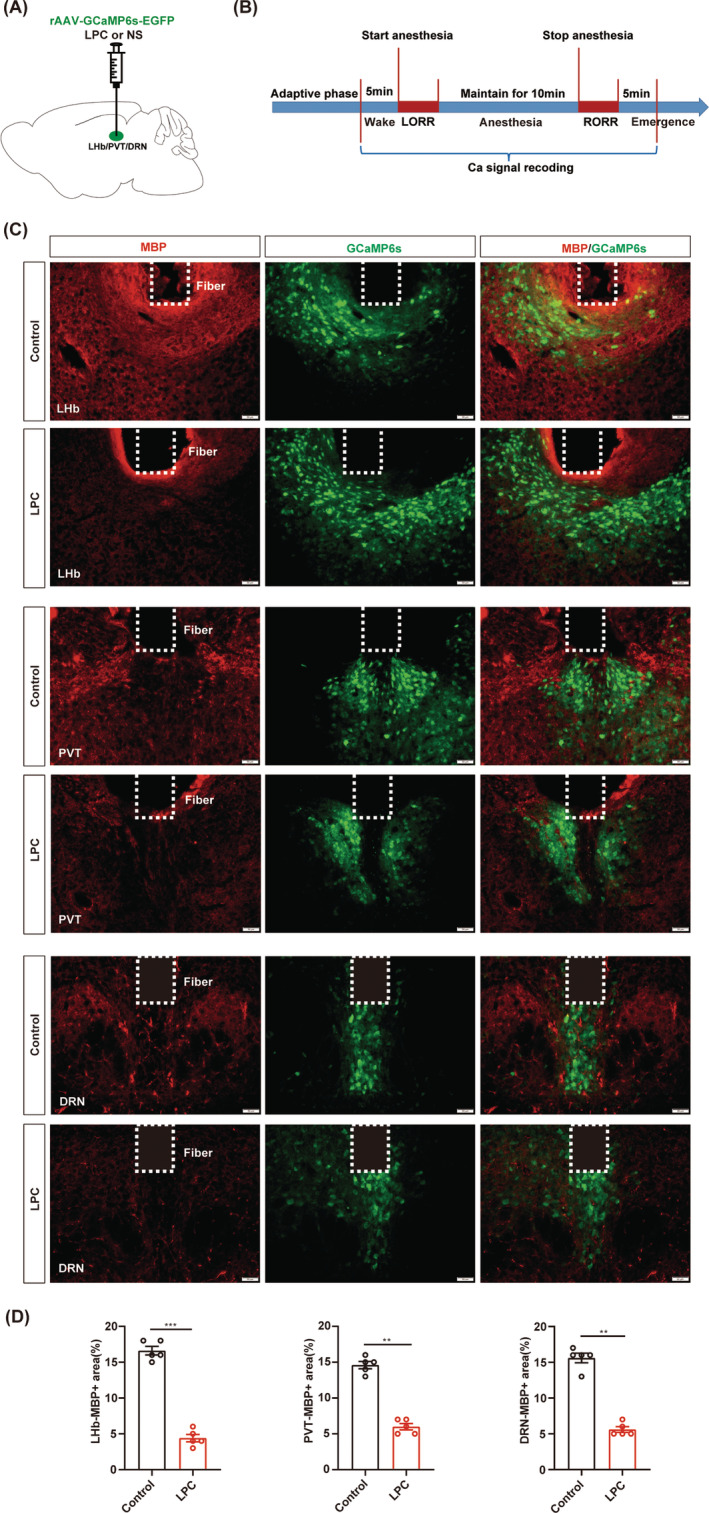
Representative expression of GCaM6Ps virus in the LHb, PVT and DRN‐demyelinated mice. (A) Schematic of LPC and virus injections. (B) Flow chart for calcium fiber photometry recording during 1.4% isoflurane anesthesia. (C) Representative images of MBP expression (red) and GCaM6Ps virus expression (green) after demyelination in LHb (top), PVT (middle), and DRN (bottom), respectively. Scale bar; 50 μm. (D) Quantification of MBP+ area in the LHb, PVT and DRN of control and LPC groups. **p < 0.01, ***p < 0.001, unpaired Student's *t*‐test. LHb, the lateral habenula; PVT, the paraventricular nucleus; DRN, the dorsal raphe nucleus.

**FIGURE 7:**
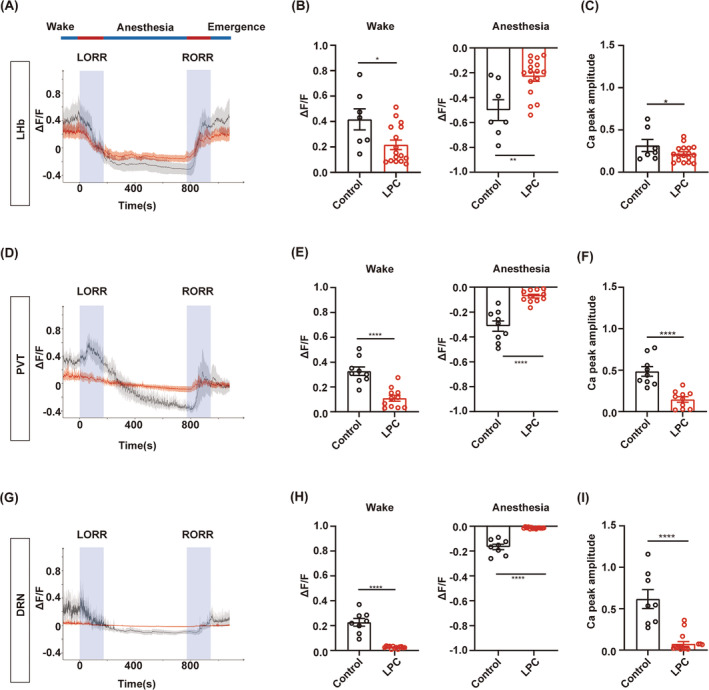
Calcium fiber photometry recording in the LHb, PVT and DRN‐demyelinated mice during isoflurane anesthesia. (A, D, G) Averaged traces of calcium signals in LPC (red) and control (black) groups under 1.4% isoflurane anesthesia in the LHb, PVT, DRN‐demyelinated tests, respectively. (B, E, H) The ΔF/F statistical plot of calcium signal during WAKE (left) and anesthesia (right) in the LPC and control groups under 1.4% isoflurane anesthesia in the LHb (*n* = 16 in the LPC group, *n* = 7 in the control group), PVT (*n* = 11 in the LPC group, *n* = 9 in the control group), DRN (*n* = 15 in the LPC group, *n* = 8 in the control group) demyelinated tests, respectively. (C, F, I) The ΔF/F statistical quantification of calcium peak amplitude in the LPC and control groups under 1.4% isoflurane anesthesia in the LHb (top), PVT (middle), DRN (bottom) demyelinated tests, respectively. *p < 0.05, **p < 0.01, ****p < 0.0001, unpaired Student's *t*‐test. LHb, the lateral habenula; PVT, the paraventricular nucleus; DRN, the dorsal raphe nucleus.

Next, we conducted real‐time calcium fiber recordings to monitor the variation of neuronal activity during isoflurane anesthesia in the LHb, PVT or DRN‐demyelinated mice (Figure 6A,B). Myelin loss was confirmed by the MBP expression and the fluorescence signal of GCaMP6s virus showed the fiber photometry recording site in the LHb, PVT and DRN, respectively (Figure 6C,D). The averaged traces plot of calcium signal revealed that the fluorescence intensity of calcium signals in the LPC group was lower than that observed in the control group during the wake period before isoflurane anesthesia (Figure 7A,D,G). The statistical analysis revealed a significant reduction in the calcium signal peak in the LPC group compared to those in the control group, indicating that demyelination leads to a decline in neuronal basal activity (Figure 7C,F,I). During isoflurane anesthesia, the amplitude of changes in calcium signal fluorescence intensity in the LPC group was significantly lower than that in the control group, which proved that the response ability of neurons to isoflurane stimulation after demyelination was greatly reduced, and the neuronal activity was barely altered during the process of isoflurane anesthesia (Figure 7A,B,D,E,G,H). These findings illustrate that the involvement of the myelin in regulating isoflurane anesthesia may be mediated through the impact on neuronal activity.

### 3.7 Demyelination in the LHb and PVT blunts the effects of neuron activation on isoflurane anesthesia

**FIGURE 8:**
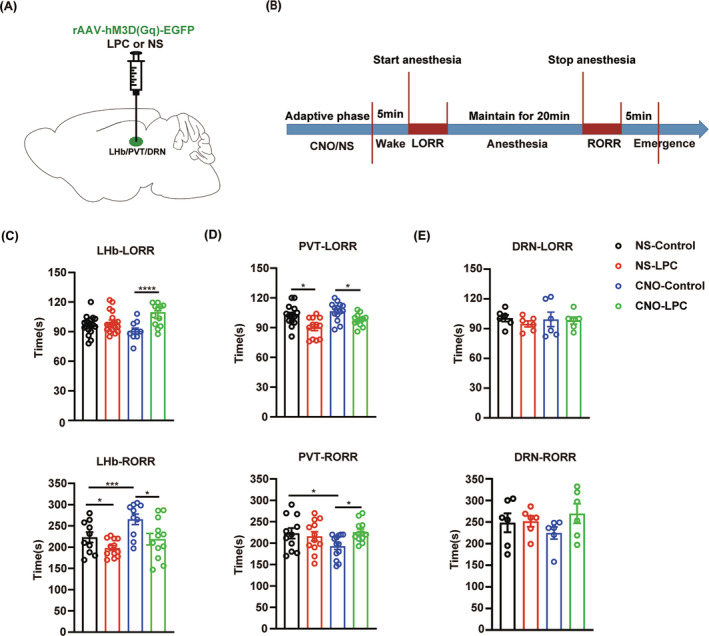
Demyelination inhibits the effect of neuronal activation on isoflurane anesthesia in the LHb and PVT. (A) Schematic of LPC and virus injections. (B) Flow chart for behavioral recording under isoflurane anesthesia. (C) Bar graphs of LORR and RORR time in the NS‐Control, NS‐LPC, CNO‐Control and CNO‐LPC groups of LHb‐demyelinated experiment during 1.4% isoflurane anesthesia (*n* = 10 in each group). (D) Bar graphs of LORR and RORR time in the NS‐Control, NS‐LPC, CNO‐Control and CNO‐LPC groups of PVT‐demyelinated experiment during 1.4% isoflurane anesthesia (*n* = 10 in each group). (E) Bar graphs of LORR and RORR time in the NS‐Control, NS‐LPC, CNO‐Control and CNO‐LPC groups of DRN‐demyelinated experiment during 1.4% isoflurane anesthesia (*n* = 6 in each group). *p < 0.05, ***p < 0.001, ****p < 0.0001, two‐way ANOVA. LHb, the lateral habenula; PVT, the paraventricular nucleus; DRN, the dorsal raphe nucleus.

Finally, we tested the effect of demyelination in the LHb, PVT or DRN on isoflurane anesthesia when we activated the neurons in the corresponding nucleus by chemogenetic approaches (Figure 8A,B). In the LHb, hM3D(Gq)‐mediated neuron activation showed a prolonged RORR time, while LPC‐induced demyelination blocked this effect (Figure 8C, Figure S2A,B). Moreover, demyelination in the PVT showed similar effect on isoflurane anesthesia after chemogenetic activation of the PVT neurons (Figure 8D, Figure S2C,D). However, no influence on isoflurane anesthesia was observed in the DRN‐demyelinated mice after chemogenetic activation of the DRN neurons (Figure 8E, Figure S2E,F). Taken together, our findings demonstrated that myelin in the LHb and PVT regulates the progress of isoflurane anesthesia through their impact on neuronal activity.

## 4 DISCUSSION

Previous studies on the mechanism of GA‐induced unconsciousness were primarily focused on the roles of neurons and neural circuits. However, the precise mechanisms of GA remain elusive. Several recent studies have shown that microglia in the CNS is involved in the GA modulation, suggesting the critical roles and mechanisms of GA modification beyond neurons.^3^, ^15^ In the present study, we revealed that CPZ‐induced demyelination in mice exhibited an extended recovery time following isoflurane anesthesia, suggesting the involvement of myelin in the regulation of GA. We further discovered that the involvement of the myelin within the distinct nuclei in regulating GA is mediated by its impact on neuronal activity. The significance of the myelin has been widely acknowledged in numerous physiological and pathological processes; however, its effect on GA regulation is remains unsure. Our study not only enhances the understanding of myelin functions in the CNS but also provides a new direction to clarify the precise neural mechanisms underlying GA.

Various studies have demonstrated the essential roles of myelin in ensuring optimal sleep quality, while also highlighting a significant association between diminished myelin content in the frontal lobe and compromised sleep quality among individuals with mental disorders.^43^, ^44^ However, whether and how myelin modulates GA has not been reported. Our study demonstrates the involvement of myelin in regulating isoflurane anesthesia through a CPZ‐induced demyelination model. The gamma oscillations (γ oscillation) in the brain are the high‐frequency (25‐100 Hz) brain waves that are believed to play a crucial role in the integration of brain information and cognitive function.^45^ Studies on the effect of anesthetics on γ oscillation power have shown that propofol and ketamine can increase it, while dexmedetomidine and high concentrations of inhaled anesthetics decrease it.^46^, ^47^, ^48^, ^49^, ^50^, ^51^ The responses of γ oscillations to different anesthetics may vary, however, the disruption of γ oscillations is inevitably associated with a certain impact on the efficacy of GA. The latest research has revealed that demyelination disrupts the synchronicity of γ oscillations in slice cultures of parvalbumin (PV) neurons.^52^ We speculate that the dual effect of demyelination and isoflurane anesthesia on γ oscillations is one reason for the significantly prolonged emergence time in the CPZ group. There are two potential explanations for the results of our study that γ oscillations in cortical EEG after demyelination within nuclei were not significantly changed. First, the cortical EEG captures the collective electrical activity of the entire cortex and may not accurately reflect neuronal activity within the nucleus. Second, compensatory mechanisms in other brain regions could be at play. To determine the effects of demyelination on neuronal electrical signals within nuclei, invasive acquisition techniques such as single neuron spikes and local field potentials (LFPS) are required.

We discovered that myelin in specific nuclei plays distinct roles in regulating isoflurane anesthesia. Demyelination in the LHb and PVT impairs the original function of these nuclei during isoflurane anesthesia. By comparing the results of neuron depletion, we observed consistent effects of myelin and neurons on isoflurane anesthesia in the LHb and PVT; however, their action processes exhibited variations. The precise mechanism underlying these differences remain unclear, with potential factors including differences in the functional structure of both myelin and neurons, as well as time‐specific processes through which they participate in anesthesia. The myelin sheath is distributed along the long axon of neurons, and it should be noted that within a nucleus, the myelin may not only consist of the axons from the certain neurons in the local nucleus but also the axons projecting from other nuclei. Therefore, demyelination occurring within the nucleus has broader implications beyond the individual nucleus. To elucidate the precise mechanisms of GA regulation between myelin and neurons in a certain nucleus, it may be necessary to clarify the origin of the myelin in the nucleus. The utilization of f‐MOST technology may partially address this issue.

In our study, myelin in the DRN exhibited different influence on isoflurane anesthesia compared to the neurons in the DRN. The different results may be due to the complex and interactive types of the DRN neurons, suggesting that the underlying mechanism could be related to neuronal activity. Subsequently, we substantiated our hypothesis by c‐Fos staining and in vivo optical fiber calcium imaging, which revealed that demyelination significantly attenuated neuronal activity in the awake mice, and caused the failure of neuronal response to isoflurane anesthesia. The inhibition of neuronal activity by demyelination is linked to the functional impairment of the myelin. The main function of CNS myelin is to provide energy support for axons and ensure accurate and rapid conduction of action potentials (AP).^53^, ^54^ Demyelination in multiple sclerosis (MS) patients and animal models results in axonal mitochondrial dysfunction, morphological alterations, and inadequate energy supply.^55^, ^56^, ^57^ Overall, myelin loss displays the detriment to energetic support of axons, with a consequent reduction in neuronal activity. In addition, myelin defect also induces abnormal conduction of neuronal electrical activity. Hypomyelination in the brain showed significant impairments in the conduction velocity, repeatability, and precision of AP, thereby deteriorating the higher‐order functions and information transmission in the CNS.^58^ The consciousness change, as a crucial target of general anesthetics, also represents a significant higher‐level function of the brain. Our data represent the essential roles of proper myelination in the regulation of isoflurane mediated consciousness change. The possible underlying mechanism is that demyelination causes destructive energy metabolism of the neurons and abnormal conduction of AP, which prevents the neuron activation and the conduction of nerve circuits. As a result, neurons not only lose their ability to regulate GA process but also fail to response to general anesthetics, including isoflurane. The intricate interactions between neurons and myelin may modify the action of nuclei or neural circuits that have been identified to be critical for higher‐level functions, as well as neuronal responses to GA. To clarify the precise mechanisms of GA, the roles of myelin cannot be ignored. Our study not only highlight the significance of myelin in regulating GA but also contribute a novel insight into further revealing the neural mechanisms underlying GA.

## 5 CONCLUSION

Myelin in the entire brain or within the distinct neural nucleus plays an essential role in regulating the process of isoflurane anesthesia. And the possible underlying mechanism is the modification of neuronal activity by myelin integrity during GA.

## AUTHOR CONTRIBUTIONS

All authors contributed to the experimental conception and design of the study. Material preparation, data collection, and analyses were performed by Xu Wang, Rulan Yi, Ning Zhang, Xiaoling Liang, Fuwang Zhong, Yali Lu, Wenjia Chen, Tian Yu, Linyong Zhang, Haiying Wang and Liang Zhou. Xu Wang, Rulan Yi and Ning Zhang generated all the mouse models. The manuscript was written by Tian Yu, Haiying Wang and Liang Zhou, and all authors commented on the previous versions of the manuscript. All authors have read and approved the final manuscript.

## FUNDING INFORMATION

This work was supported by the National Natural Science Foundation of China (grants 82060224 to L.Z.), the Science and Technology Program of Guizhou Province (grants Qian Comprehensive Basic Science [2020]1Y088, [2017]5733‐066, [2020]‐002 to L.Z.), the Joint Bidding Project by Zunyi City and Zunyi Medical University (ZSKH‐HZ [2023]‐232 to L.Y.Z.), and PhD Research Startup Foundation of Zunyi Medical University (F‐958 to L.Z.).

## CONFLICT OF INTEREST STATEMENT

The authors declare that they have no conflicts of interest in this work.

## Supporting information


Data S1.

## Data Availability

The data that support the findings of this study are available from the corresponding author upon reasonable request.
