# Supplementary material for: Myelin modulates the process of isoflurane anesthesia through the regulation of neural activity
Source: CNS Neurosci Ther. 2024 Aug 13;30(8):e14922. doi: 10.1111/cns.14922 (PMC11322027; doi:10.1111/cns.14922)
Supplement: Supplementary file 1 — Data S1. [file CNS-30-e14922-s001.pdf]

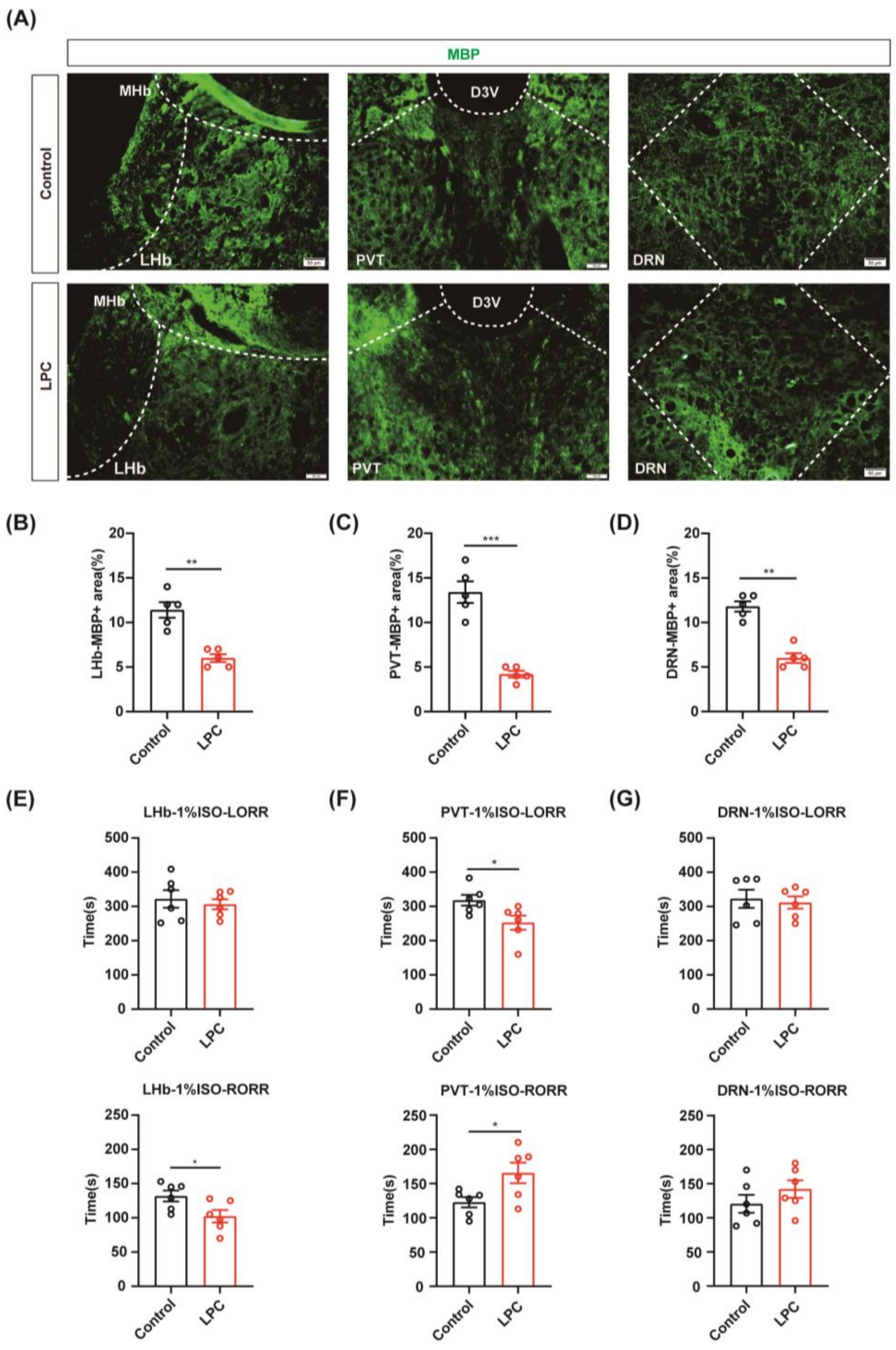

FIGURE S1. Effect of demyelination in the LHb, PVT and DRN on 1% isoflurane anesthesia. (A) Representative images of MBP staining (green) in the LHb, PVT and DRN of the LPC and control groups, respectively. Scale bar; 50 μm. (B) (C) (D) Quantification of MBP+ area in the LHb, PVT and DRN, respectively. (E) (F) (G) Bar graphs of LORR and RORR time in the LHb, PVT and DRN of the LPC and control groups during 1% isoflurane anesthesia, respectively (n = 6 in each group). **P <0.05*, ***P <0.01*, ****P <0.001*, unpaired Student's t test.

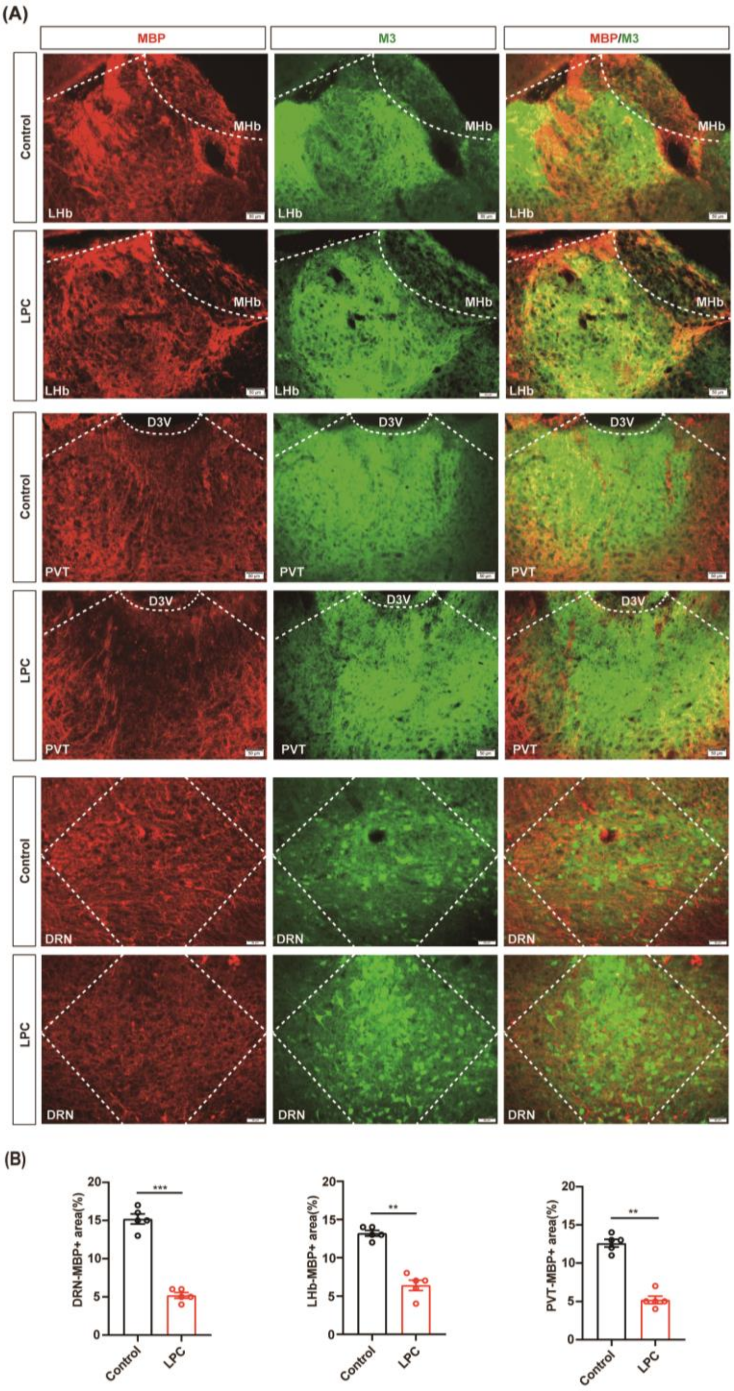

FIGURE S2. Expression of hM3D(Gq) virus in the LHb, PVT and DRN-demyelinated mice. (A) MBP (red) and hM3D(Gq) virus expression (green) after demyelination in the LHb, PVT, and DRN, respectively. Scale bar; 50 μm. (B) Quantification of MBP+ area in the LHb, PVT and DRN of control and LPC groups (n = 6 in each group). ***P* <0.01, ****P* <0.001, unpaired Student's t-test.
